# Quality of life and illness perceptions in patients with breast cancer using a fasting mimicking diet as an adjunct to neoadjuvant chemotherapy in the phase 2 DIRECT (BOOG 2013–14) trial

**DOI:** 10.1007/s10549-020-05991-x

**Published:** 2020-11-11

**Authors:** Rieneke T. Lugtenberg, Stefanie de Groot, Ad A. Kaptein, Maarten J. Fischer, Elma Meershoek-Klein Kranenbarg, Marjolijn Duijm-de Carpentier, Danielle Cohen, Hiltje de Graaf, Joan B. Heijns, Johanneke E. A. Portielje, Agnes J. van de Wouw, Alex L. T. Imholz, Lonneke W. Kessels, Suzan Vrijaldenhoven, Arnold Baars, Marta Fiocco, Jacobus J. M. van der Hoeven, Hans Gelderblom, Valter D. Longo, Hanno Pijl, Judith R. Kroep, on behalf of the Dutch Breast Cancer Research Group (BOOG)

**Affiliations:** 1 Department of Medical Oncology, Leiden University Medical Center, Albinusdreef 2, Leiden, P.O. Box 9600, 2300 RC Leiden, The Netherlands; 2 Department of Medical Psychology, Leiden University Medical Center, P.O. Box 9600, 2300 RC Leiden, The Netherlands; 3 Department of Surgery, Leiden University Medical Center, P.O. Box 9600, 2300 RC Leiden, The Netherlands; 4 Department of Pathology, Leiden University Medical Center, P.O. Box 9600, 2300 RC Leiden, The Netherlands; 5 Department of Medical Oncology, Medical Center Leeuwarden, P.O. Box 888, 8901 NR Leeuwarden, The Netherlands; 6 Department of Medical Oncology, Amphia, P.O. Box 90157, 4800 RL Breda, The Netherlands; 7 Department of Medical Oncology, Haga Hospital, Den Haag, P.O. Box 40551, 2504 LN The Hague, The Netherlands; 8 Department of Medical Oncology, Viecuri, 5912 BL Venlo, The Netherlands; 9 Department of Medical Oncology, Deventer Hospital, P.O. Box 5001, 7416 SE Deventer, The Netherlands; 10 Department of Medical Oncology, Noordwest Hospital Group, Location Alkmaar, P.O. Box 501, 1815 JD Alkmaar, The Netherlands; 11 Department of Medical Oncology, Hospital Gelderse Vallei, 6710 HN Ede, The Netherlands; 12 Department of Medical Statistics and Bioinformatics, Leiden University Medical Center, P.O. Box 9600, 2300RC Leiden, The Netherlands; 13 Department of Biological Sciences, Longevity Institute, School of Gerontology, University of Southern California, Los Angeles, CA 90089 USA; 14 IFOM FIRC Institute of Molecular Oncology, Via Adamello 16, Milan, Italy; 15 Department of Endocrinology, Leiden University Medical Center, P.O. Box 9600, 2300 RC Leiden, The Netherlands; 16 BOOG Study Center, P.O. Box 9236, 1006 AE Amsterdam, The Netherlands

**Keywords:** Quality of life, Illness perceptions, Breast cancer, Chemotherapy, Short-term fasting, Fasting mimicking diet, Distress thermometer

## Abstract

**Purpose:**

In the phase II DIRECT study a fasting mimicking diet (FMD) improved the clinical response to neoadjuvant chemotherapy as compared to a regular diet. Quality of Life (QoL) and illness perceptions regarding the possible side effects of chemotherapy and the FMD were secondary outcomes of the trial.

**Methods:**

131 patients with HER2-negative stage II/III breast cancer were recruited, of whom 129 were randomly assigned (1:1) to receive either a fasting mimicking diet (FMD) or their regular diet for 3 days prior to and the day of neoadjuvant chemotherapy. The European Organisation for Research and Treatment of Cancer (EORTC) questionnaires EORTC-QLQ-C30 and EORTC-QLQ-BR23; the Brief Illness Perception Questionnaire (BIPQ) and the Distress Thermometer were used to assess these outcomes at baseline, halfway chemotherapy, before the last cycle of chemotherapy and 6 months after surgery.

**Results:**

Overall QoL and distress scores declined during treatment in both arms and returned to baseline values 6 months after surgery. However, patients’ perceptions differed slightly over time. In particular, patients receiving the FMD were less concerned and had better understanding of the possible adverse effects of their treatment in comparison with patients on a regular diet. Per-protocol analyses yielded better emotional, physical, role, cognitive and social functioning scores as well as lower fatigue, nausea and insomnia symptom scores for patients adherent to the FMD in comparison with non-adherent patients and patients on their regular diet.

**Conclusions:**

FMD as an adjunct to neoadjuvant chemotherapy appears to improve certain QoL and illness perception domains in patients with HER2-negative breast cancer.

Trialregister

ClinicalTrials.gov Identifier: NCT02126449.

**Electronic supplementary material:**

The online version of this article (10.1007/s10549-020-05991-x) contains supplementary material, which is available to authorized users.

## Introduction

Short-term fasting (STF) during cancer treatment has attracted increasing attention since the first report of benefits in mice in 2008 [1]. Indeed, in rodents, fasting limits tumor proliferation and enhances the sensitivity of tumor cells to cancer therapies, while simultaneously protecting healthy cells against its toxic effects [2–4]. These experimental benefits triggered a number of small clinical trials exploring the potential of STF during cancer treatment [5, 6]*,* which suggested similar effects in humans*.*

Water-only fasting is difficult to sustain and may have adverse effects associated with energy- and/or micronutrient deficiencies. Fasting mimicking diets (FMD) are designed to mimic the physiologic effects of water-only fasting, while offering minimally required (micro)nutrients [4, 7]. These diets are plant-based and primarily comprise complex carbohydrates and healthy fats, while simple carbohydrates are virtually absent and protein content is low. We recently reported that an FMD, as compared to regular diet, enhanced the radiological as well as the pathological tumor response to chemotherapy in women with HER2-negative breast cancer [8]. Despite omitting dexamethasone prior to chemotherapy in the FMD arm, grade III/IV toxicity was similar in both study arms and chemotherapy-induced DNA damage in lymphocytes was less in patients receiving the FMD, suggesting that the diet simultaneously limits adverse effects in healthy cells.

Cancer, as well as its treatment, significantly reduces the quality of life QoL of patients [9, 10]. Individuals construct cognitive and emotional representations of an illness (i.e., illness perceptions) as an adaptive mechanism [11]. Illness perceptions can be used to explain behavior following heart attacks, the response to cancer screening or how patients cope with cancer treatment [12]. The Brief Illness Perception Questionnaire (BIPQ) is a validated, widely used instrument to assess patients’ cognitions and emotions about an illness, a received or proposed treatment or future perspectives [12]. Negative illness perceptions in patients with cancer, or other chronic diseases, have been associated with worse health outcomes, such as higher mortality rates, more severe symptom burden and poorer treatment adherence [13–16]. Furthermore, patients with cancer and negative illness perceptions have been reported to experience lower quality of life and more physical distress [17–19].

It is unknown if STF or FMDs affect cancer patients’ QoL and illness perceptions. Previous studies suggest that STF is safe, well tolerated, and perhaps even associated with improved QoL [20–22]. Indeed, QoL increased without any serious side effect in more than 2000 subjects with chronic illness and pain syndromes, who used a very low-calorie diet of 350 kcal per day for 7 days [23]. A small randomized cross-over trial with 34 patients evaluated the effect of STF on QoL in patients with breast cancer and ovarian cancer treated with chemotherapy. STF enhanced tolerance to chemotherapy, while QoL was less compromised and fatigue was reduced [22]. Little data is available about patients’ motivations and perceptions of fasting during cancer treatment. Interviews conducted in a group of 16 patients with breast cancer showed that fasting gave them a greater sense of control over their treatment [24]. If patients are randomized to receive an FMD they can contribute personally to their treatment. This may lead to more active involvement and different illness perceptions regarding treatment and its possible side effects.

The multicenter, open label, phase II randomized DIRECT study was conducted to evaluate the impact of an FMD on toxicity as well as on the response to neoadjuvant chemotherapy in patients with HER2-negative breast cancer [8]. QoL and illness perceptions regarding the possible side effects of chemotherapy and the FMD were secondary outcomes of the DIRECT trial. Our hypothesis is that patients on an FMD would experience less toxic side effects from their treatment, resulting in better QoL, less distress and more positive perceptions towards possible side effects, compared to patients on a regular diet.

## Methods

### Study design and treatment

The detailed study design has been previously reported in Nature Communications [8]. In brief, the DIRECT trial was a multicenter, open label, phase II trial randomizing between an FMD or regular diet for 3 days prior to and the day of neoadjuvant chemotherapy and before surgery in women with HER2-negative breast cancer. The FMD is a 4-day plant-based low amino acid substitution diet, consisting of soups, broths, liquids vitamin tablets and tea. Calorie content declined from day 1 (~1200 kcal), to days 2–4 (~200 kcal) (supplementary material). All patients provided informed consent prior to start of chemotherapy and randomization. This study (NCT02126449) was conducted in accordance with the Declaration of Helsinki (October 2013) and approved by the Ethics Committee of the Leiden University Medical Center in agreement with the Dutch law for medical research involving human subjects.

### Monitoring adherence

On the day of each cycle of chemotherapy (prior to drug administration), fasting values of glucose, insulin and IGF-1 were determined in plasma for all patients in both study arms and ketone bodies in an urine portion. Also during this visit adherence to FMD or normal diet was noted by the oncologist or research nurse based on self-reports of patients.

### Patient-reported quality of life, illness perceptions and burden

Outcomes were assessed at baseline (QoL and Illness Perception), halfway chemotherapy (QoL and Distress), before the last cycle of chemotherapy (QoL, Distress and Illness Perception) and 6 months after surgery (QoL and Distress) to explore long-term effects of the intervention. The amount of distress caused by treatment was not measured at baseline, since patients had not receive treatment at that time.

#### Quality of life

Global QoL, functioning and symptoms were assessed with the European Organisation for Research and Treatment of Cancer QoL Questionnaire Core 30 (EORTC-QLQ-C30) and Breast Cancer Questionnaire (QLQ-BR23). The EORTC-QLQ-C30 includes 30 items covering five functional scales; ten symptom scales or single items and one global health status scale [25]. The EORTC-QLQ-BR23 collects disease-specific data. It comprises 23 items, divided into four functioning scales and four symptom scales. This questionnaire is widely used to assess breast cancer-related problems [26]. The items covering “breast symptoms and arm symptoms” were excluded in our study, as the trial concerned neoadjuvant therapy and patients did not have surgery yet.

In accordance with the scoring manual linear transformed scores were computed for the QLQ-C30 and QLQ-BR23 scales for each assessment time point [27]. Differences of at least 10 points on the scales/items were defined as the threshold for minimum of clinically significant difference [28].

#### Distress

Patients were asked to rate their overall distress caused by their treatment on a visual analog scale (a thermometer). The Distress Thermometer (DT) is developed and validated for evaluation of distress in patients with cancer [29]. The DT is a single-item instrument that relates to the level of distress (range 0–10) a patient has experienced in the past week. A score of ≥5 was the cut-off for clinically relevant distress, based on a Dutch validation study [29].

#### Illness perception

Illness perceptions about the possible side effects of chemotherapy and effectiveness of an FMD were assessed with the Brief Illness Perception Questionnaire (BIPQ). The BIPQ consists of eight questions that measure eight dimensions of illness perceptions in the following order: Understanding (how well do you feel you understand your illness), Consequences (how much does your illness affect your life), Timeline (how long do you think your illness will last), Personal Control (how much control do you feel you have over your illness), Treatment Control (how much do you think your treatment can help your illness), Identity (how much do you experience symptoms from your illness), Concern (how concerned are you about your illness), and Emotional Representation (how much does your illness affect you emotionally). For this study, the word “illness” was replaced with “possible side effects of chemotherapy” and the word “treatment” was replaced with “a fasting mimicking diet”. Answers were given on a scale ranging from 0 to 10 [30]. Higher scores represent more negative illness perception, except for understanding, personal control and treatment control.

### Statistical analyses

The sample size was based on the primary study endpoint of this phase II trial, grade III/IV toxicity. Patients were evaluable for analysis if they completed the set of baseline questionnaires and at least one of the consecutive questionnaires. The questionnaire adherence per cycle was measured as the percentage of patients completing each instrument.

A two-sided Fisher exact rest was used to compare the proportion of adherent patients for each questionnaire between randomization groups. Continuous variables with a normal distribution were expressed as mean value and standard deviation. Comparison of baseline characteristics was performed using the Fisher exact test for categorical variables and the two-tailed Student’s *t* test for continuous variables. The effect of the FMD on the different QoL scales and distress were estimated using linear mixed models, with an unstructured covariance matrix including treatment, time and the interaction between treatment and time. For each scale, all scores over time were used as the dependent outcome in the models. The baseline measures: clinical stage, hormonal status, body mass index and type of chemotherapy were entered in the model as covariates. With the use of a mixed model, we can deal with correlated structure in the present data, without adjustments for multiple comparisons. Because the measurements of illness perceptions consisted of only two time points, the effect of the FMD on BIPQ scores was estimated with a linear regression model with the same covariates entered in the mixed models. The analyses were performed according to the intention-to-treat principle. A post hoc, exploratory per-protocol analysis was done to explore the effects of the FMD on QoL, distress and illness perceptions. Patients who were adherent to the FMD for at least half of the cycles were compared with those who were less adherent, and with the adherent control patients (i.e., the patients in the control group who did not fast on their own initiative). All tests were 2-tailed with a significance level of 0.05. All data were analyzed using IBM SPSS Statistics for Windows (Version 25.0. Armonk, NY: IBM Corp).

## Results

### Patient characteristics

**Fig. 1:**
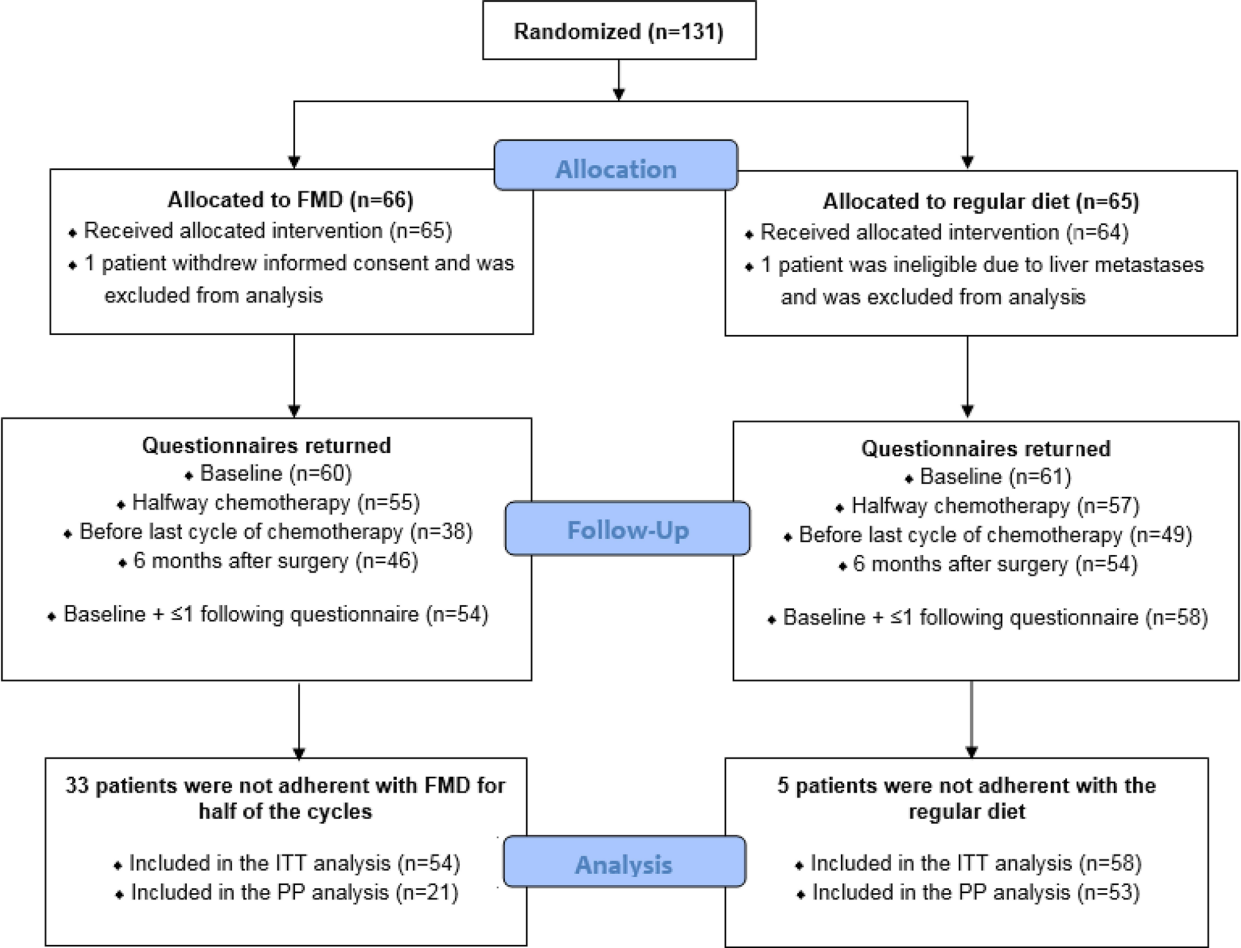
Flow diagram. *FMD *fasting mimicking diet, *ITT* intention-to-treat, *PP* per-protocol

From February 2014 to January 2018, 131 patients from 11 centers from the Dutch Breast Cancer Research Group (BOOG) were randomized. One patient withdrew informed consent before starting with chemotherapy and one patient was ineligible because of liver metastasis, which were diagnosed the day after randomization. Of the remaining 129 patients, 65 received the FMD as an adjunct to the standard chemotherapy and 64 used their regular diet (Fig. 1). Patients’ characteristics were well balanced between the two study arms (Table 1).

**Table 1:** Patient characteristics

	FMD (*n* = 65)	Regular diet (*n* = 64)
Median age (range), years	49.0 (31–71)	51.0 (27–71)
Median body mass index (range), kg/m^2^	25.7 (19.8–41.2)	26.0 (19.7–39.0)
WHO status		
Grade 0	61 (93.8%)	60 (93.8%)
Grade 1	3 (4.6%)	4 (6.3%)
Unknown	1 (1.5%)	0 (0%)
Menopausal status		
Pre/peri	38 (58.5%)	31 (48.4%)
Post	27 (41.5%)	31 (48.4%)
Unknown	0 (0%)	2 (3.1%)
T classification		
T1	5 (7.7%)	6 (9.4%)
T2	42 (64.6%)	41 (64.1%)
T3	17 (26.2%)	15 (23.4%)
T4	1 (1.5%)	2 (3.1%)
N classification		
N0	29 (44.6%)	33 (51.6%)
N1	28 (43.1%)	26 (40.6%)
N2	7 (10.8%)	4 (6.3%)
N3	1 (1.5%)	1 (1.6%)
Clinical stage		
I (ineligible)	0 (0%)	1 (1.6%)
II	51 (78.5%)	48 (75.0%)
III	14 (21.5%)	15 (23.4%)
HR status		
HR−	14 (21.5%)	8 (12.5%)
HR+	51 (78.5%)	56 (87.5%)
Chemotherapy regimen		
AC-T	52 (80.0%)	47 (73.4%)
FEC-T	13 (20.0%)	17 (26.6%)
Grade (BR)		
I	2 (3.1%)	2 (3.1%)
II	43 (66.2%)	42 (65.6%)
III	20 (30.8%)	19 (29.7%)
Unknown	0 (0%)	1 (1.6%)
Tumortype		
Ductal	53 (81.5%)	49 (76.6%)
Lobular	9 (13.8%)	13 (20.3%)
Other	3 (4.6%)	2 (3.1%)

*FMD* fasting mimicking diet, *HR* hormone receptor, *AC-T* doxorubicin/cyclophosphamide followed by docetaxel,* FEC-T* fluorouracil/epirubicin/cyclophosphamide followed by docetaxel, *BR* bloom Richardson

### Adherence to the FMD

Fifty three out of 65 patients (81.5%) completed the first FMD cycle, whereas over 50% completed at least 2 FMD cycles. 22 out of 65 patients (33.8%) used the FMD for at least four cycles, and 21.5% of the patients adhered to FMD during all cycles of chemotherapy (Table 2). The main reason for non-adherence to the FMD was aversion to distinct components of the diet, perhaps induced by chemotherapy. In the regular diet group, 5 (7.8%) patients did not adhere (they decided to fast during one or more cycles of chemotherapy).

**Table 2:** Adherence to the assigned diet

	FMD (*N *= 65)	Regular diet (*N* = 64)
Adherent—all cycles and surgery		
Yes	10 (15.4%)	59 (92.2%)
No	55 (84.6%)	5 (7.8%)
Adherent—all cycles*		
Yes	14 (21.5%)	
No	51 (78.5%)	
Adherent—half of cycles**		
Yes	22 (33.8%)	
No	43 (66.2%)	
Adherent—first cycle		
Yes	53 (81.5%)	
No	11 (16.9%)	
Unknown	1 (1.5%)	
Reason for early stop FMD		
Taste	26 (51.0%)	
Nausea	10 (15.4%)	
Hunger	5 (9.8%)	
Stop chemotherapy	2 (3.9%)	
Other	8 (35.3%)	

Adherence given per group.

*FMD*: Fasting mimicking diet.

*Adherence is defined as patients complied the FMD or regular diet all cycles of their treatment arm.

**Adherence is defined as patients complied to the FMD or regular diet half of the cycles of their treatment arm. Regular diet patients were non-adherent if they were fasting for at least one cycle on their own.

### Weight changes

Patients randomized for the FMD displayed a decrease in body mass index (BMI) halfway therapy (median decrease 0.38 kg/m^2^, range −2.16 to +3.43, *P* = 0.002) and at the end of therapy (median decrease 0.33 kg/m^2^, range −2.48 to +4.81, *P* = 0.026). In the regular diet group BMI at the end of therapy was higher than at baseline (median increase 0.64 kg/m^2^, range −3.93 to +4.71, *P* = 0.006). This difference persisted 6 months after surgery (median increase of 0.56 kg/m^2^, range −2.03 to +6.17, *P* = 0.043) in patients on a regular diet, whereas the BMI of patients on an FMD did not differ from the BMI before start of chemotherapy.

### Completion of questionnaires

Questionnaire set 1 (EORTC-QLQ-C30, EORTC BR23 and the BIPQ) was completed by 121 patients (94%) before the start of chemotherapy, questionnaire set 2 (EORTC-QLQ-C30, EORTC BR23 and the DT) was completed by 112 patients (87%) halfway chemotherapy, questionnaire set 3 (EORTC-QLQ-C30, EORTC BR23, DT and the BIPQ) was completed by 87 patients (67%) before the last cycle of chemotherapy and questionnaire set 4 (EORTC-QLQ-C30, EORTC BR23 and the DT) was completed by 100 patients (78%) 6 months after surgery. Non-response to the third set of questionnaires occurred more frequently in the FMD arm (41% vs. 23%, *p* < 0.05).

### Quality of life

**Fig. 2:**
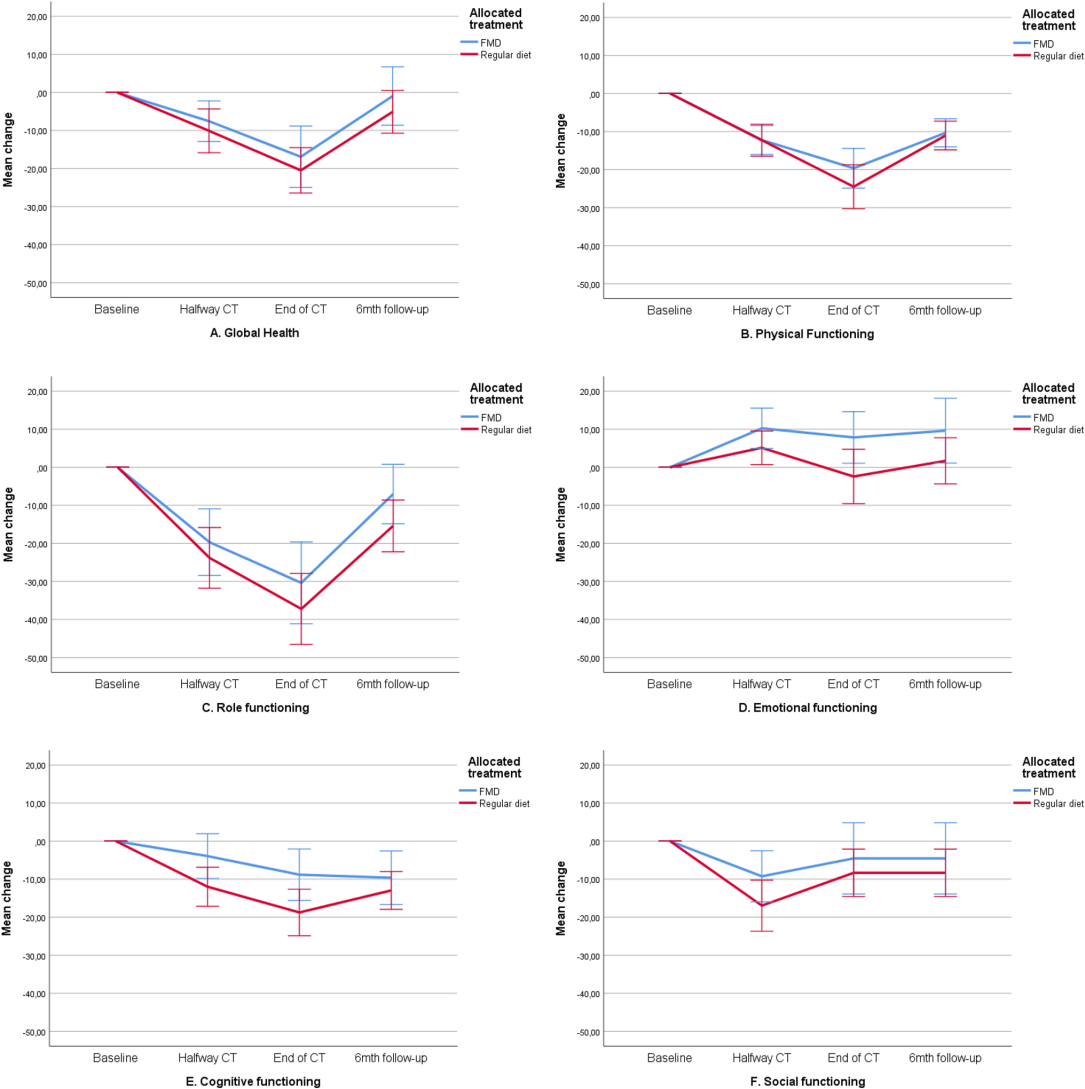

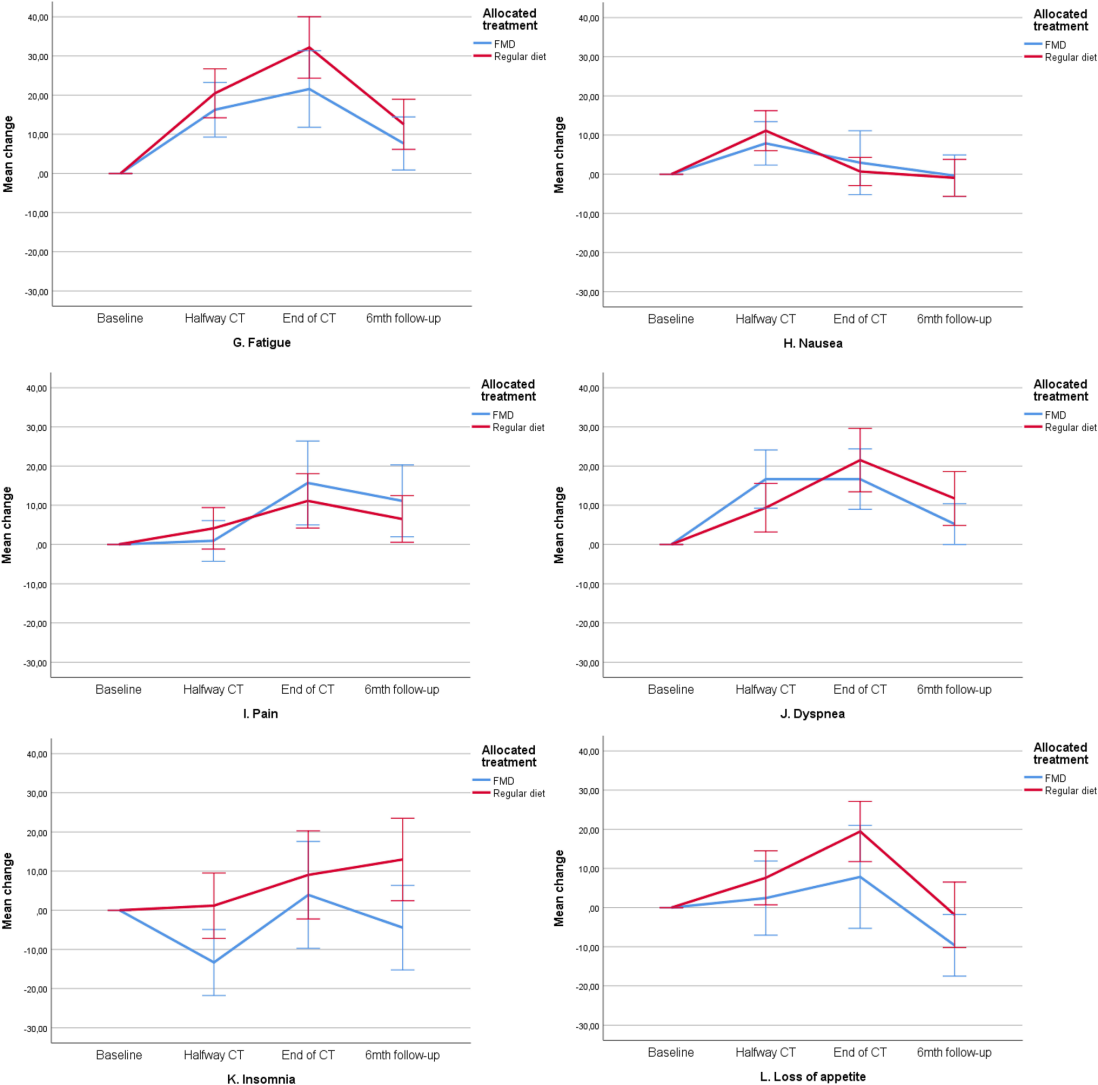

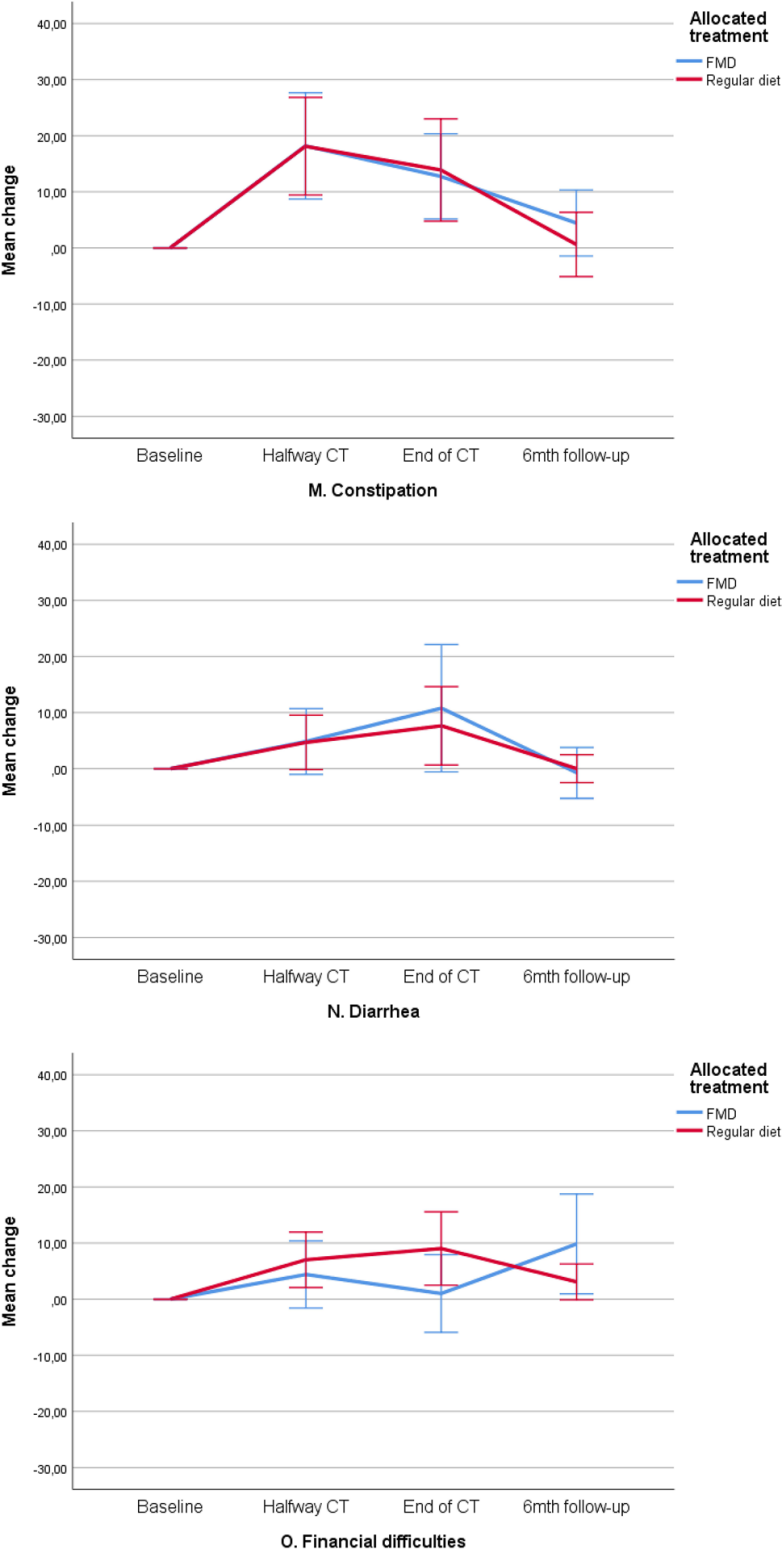
**a**–**o** Mean changes from baseline on functional and symptom scales of the EORTC-QLQ-C30. These plots show mean changes and 95% CIs calculated from the raw data; they are not model estimates, and they are not adjusted for any covariates. *CT *chemotherapy,* FMD *fasting mimicking diet, *CI* confidence interval. Lower scores on the functional scales (**a**–**f**) implicates lower quality of life, lower scores on the symptom scales (**g**–**o**) implicate better quality of life

**Fig. 3:**
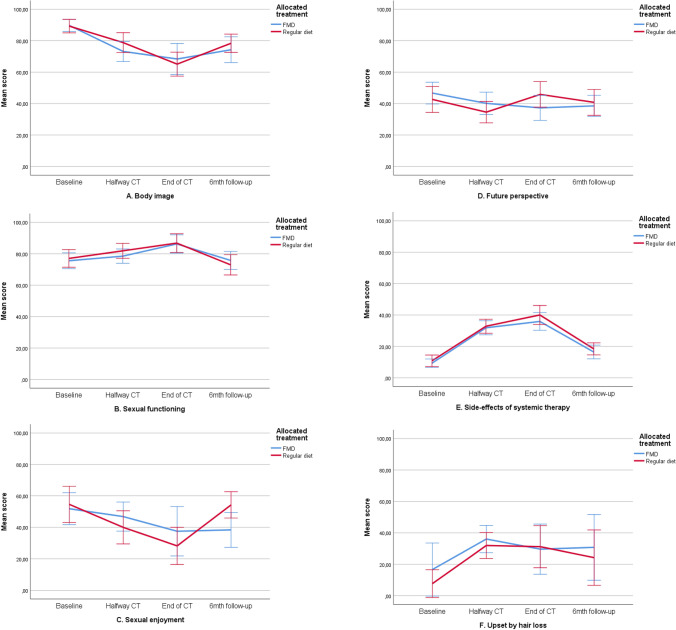
**a**–**f** Mean scores on functional and symptom scales of the EORTC-QLQ-BR23. These plots show mean scores and 95% CIs calculated from the raw data; they are not model estimates, and they are not adjusted for any covariates. Lower scores on the functional scales (**a**–**d**) implicates lower quality of life, lower scores on the symptom scales (**e**, **f**) implicate better quality of life. *CT* chemotherapy, *FMD *fasting mimicking diet, *CI* confidence interval

The mean baseline overall QLQ‐C30 and QLQ‐BR23 scale scores were similar in both treatment groups (Table 3). Scores deteriorated similarly during chemotherapy in both study arms and returned to baseline values during follow-up. Figures 2a–n, 3a–f and Table 4 present QoL scores over time in both groups.

**Table 3:** Quality of life, illness perceptions and distress scores at baseline for evaluable patients (mean, SD)

	FMD (*n* = 54)	Regular diet (*n* = 58)	*p* value
EORTC QoL-C30 domains			
Global Health	79.5 (18.72)	80.5 (19.72)	0.787
Physical functioning	96.4 (6.44)	93.7 (11.13)	0.117
Role functioning	86.7 (22.08)	90.1 (17.78)	0.372
Emotional functioning	69.9 (20.59)	75.3 (20.17)	0.163
Cognitive functioning	84.2 (18.82)	89.4 (16.72)	0.128
Social functioning	88.6 (18.26)	92.0 (13.33)	0.264
EORTC QoL-C30 symptoms			
Fatigue	23.0 (24.65)	19.0 (18.26)	0.320
Nausea	4.2 (12.92)	4.3 (11.91)	0.977
Pain	7.6 (14.98)	11.5 (16.28)	0.187
Dyspnea	3.7 (10.57)	4.6 (11.59)	0.671
Insomnia	32.7 (30.42)	26.4 (31.07)	0.280
Appetite loss	14.5 (24.65)	9.8 (18.74)	0.247
Constipation	4.85 (13.48)	6.9 (21.41)	0.547
Diarrhea	2.4 (10.84)	2.3 (10.56)	0.950
Financial difficulties	3.8 (12.51)	2.9 (9.44)	0.668
EORTC Qol-BR23 scores			
Body image	90.3 (14.23)	89.9 (16.65)	0.889
Sexual functioning	76.1 (19.18)	77.0 (22.26)	0.809
Sexual enjoyment	56.1 (31.10)	58.1 (32.17)	0.802
Future perspective	47.9 (27.04)	42.0 (31.57)	0.287
EORTC Qol-BR23 symptoms			
Systemic side effects	9.6 (10.58)	10.5 (14.25)	0.709
Upset by hair loss	33.3 (23.57)	16.7 (18.26)	0.218

	FMD (*n* = 33)	Regular diet (*n* = 47)	*p* value
BIPQ			
Understanding	7.67 (1.61)	7.13 (1.61)	0.149
Consequences	6.33 (2.04)	6.28 (1.87)	0.898
Timeline	4.45 (1.75)	4.89 (1.90)	0.297
Personal control	4.39 (1,92)	4.43 (1.93)	0.927
Treatment control	3.48 (1.79)	3.74 (2.05)	0.558
Identity	5.73 (1.88)	5.83 (1.59)	0.793
Concern	6.09 (2.28)	5.78 (1.99)	0.525
Emotional response	5.55 (2.21)	5.33 (2.26)	0.669

	FMD (*n* = 49)	Regular diet (*n* = 55)	*p* value
Distress thermometer	5.02 (2.10)	5.13 (2.18)	0.800

**Table 4:** Mean scores of QLQ-C30 and QLQ-BR23 functioning scales and symptom ratings by treatment arm

	Questionnaire time points				*p* value		
	Baseline	Halfway CT	Before last cycle of CT	6 months after surgery	Time	Randomization	Time by randomization
EORTC-QLQ-C30							
Global Health							
Regular diet	80.5	70.3	59.2	75.8	** <0.001**	0.298	0.883
FMD	79.5	71.2	61.5	78.7			
Functioning scales							
Physical functioning							
Regular diet	93.7	81.9	68.8	82.8	** <0.001**	0.316	0.562
FMD	96.4	84.0	77.0	86.2			
Role functioning							
Regular diet	90.1	66.1	52.4	74.1	** <0.001**	0.392	0.653
FMD	86.7	67.0	52.9	78.1			
Emotional functioning							
Regular diet	75.3	80.7	73.4	78.1	**0.048**	0.215	0.631
FMD	69.9	80.2	78.4	80.0			
Cognitive functioning							
Regular diet	89.4	77.2	70.5	76.9	**0.011**	0.106	0.533
FMD	84.2	80.3	75.5	74.8			
Social functioning							
Regular diet	92.0	74.9	62.8	83.6	**0.005**	0.467	0.724
FMD	88.6	78.8	68.6	83.3			
Symptoms							
Fatigue							
Regular diet	19.0	39.8	52.8	31.1	** <0.001**	0.154	0.393
FMD	23.0	39.3	43.5	28.9			
Nausea							
Regular diet	4.3	15.5	5.9	2.5	** <0.001**	0.629	0.312
FMD	4.2	12.1	7.4	3.1			
Pain							
Regular diet	11.5	15.8	22.9	17.3	** <0.001**	0.992	0.159
FMD	7.6	8.5	25.5	20.0			
Dyspnea							
Regular diet	4.6	14.0	26.4	16.0	**0.001**	0.694	**0.045**
FMD	3.7	20.6	20.6	9.6			
Insomnia							
Regular diet	26.4	26.9	37.5	38.3	** <0.001**	0.068	0.246
FMD	32.7	19.4	39.2	31.1			
Appetite loss							
Regular diet	9.8	17.5	29.9	7.4	** <0.001**	0.196	0.535
FMD	14.5	17.0	21.6	5.2			
Constipation							
Regular diet	2.3	25.1	22.2	8.0	** <0.001**	0.654	0.826
FMD	2.4	23.0	16.7	8.1			
Diarrhea							
Regular diet	6.9	7.0	10.4	1.2	** <0.001**	0.623	0.749
FMD	4.8	7.3	13.7	2.2			
Financial difficulties							
Regular diet	2.9	9.9	11.8	6.2	0.807	0.981	**0.014**
FMD	3.8	8.5	5.9	14.1			
EORTC-QLQ-BR23							
Body image							
Regular diet	89.9	78.8	65.1	78.4	** <0.001**	0.466	0.366
FMD	90.3	73.2	68.4	74.3			
Sexual functioning							
Regular diet	77.0	81.9	86.8	73.0	** <0.001**	0.815	0.292
FMD	76.1	78.5	86.3	75.8			
Sexual enjoyment							
Regular diet	58.1	44.4	43.1	55.9	**0.001**	0.554	**0.040**
FMD	56.1	49.5	42.9	48.7			
Future perspective							
Regular diet	42.0	34.5	45.8	40.7	0.184	0.579	0.085
FMD	47.9	40.1	37.3	38.5			
Symptom scales							
Side effects of systemic therapy							
Regular diet	10.5	32.8	40.0	18.5	** <0.001**	0.378	0.817
FMD	9.6	31.9	35.9	16.5			
Upset by hair loss							
Regular diet	16.7	32.6	31.1	50.0	**0.003**	0.465	0.187
FMD	33.3	36.8	31.4	40.0			
Distress thermometer							
Regular diet		5.21	6.27	6.17	0.468	0.358	0.465
FMD		5.16	5.53	5.98			

*P*-values < 0.05 were considered significant

*P time* changes of QoL scores over time, *P randomization* differences in QoL between the two treatment groups (FMD vs. regular diet), *P time by randomization* different effects between treatment groups over time, *CT* chemotherapy, *FMD* fasting mimicking diet.

#### Global health status

During treatment, the global health status scale deteriorated significantly in both study arms (*p* < 0.01) (Fig. 2a) and returned to baseline at follow-up (6 months after surgery). Similar patterns were observed in the per-protocol analysis, without any difference between adherent and non-adherent patients (supplementary material).

#### Functional scales of the QLQ-C30

Physical, role, and cognitive functioning scores declined clinically and statistically significantly in both arms during treatment (*p* < 0.01), with the lowest scores at the end of chemotherapy (Fig. 2b–f). Deterioration of social functioning was statistically significant, but not clinically relevant in either group (a decrease in score of <10; Fig. 2f). Patients reported significant improvement of emotional functioning in both arms over time (*p* < 0.05) (Fig. 2d). In the per-protocol analyses, better scores were observed in all five functional scales (physical, role, emotional, cognitive and social functioning) in patients adherent to the FMD in comparison with non-adherent patients and patients on a regular diet (supplementary material).

#### Symptom scales of the QLQ-C30

In both arms, patients reported clinically relevant and significant worsening of fatigue, pain, dyspnea, loss of appetite and constipation in the course of treatment (*p* < 0.01) (Fig. 2g–o). Patients following the FMD tended to have better scores on insomnia (Fig. 2k, *p* = 0.068). In both groups, patients reported significant worsening of nausea in the course of treatment, but in the FMD group the difference was not clinically relevant (an increase in nausea score of <10; Fig. 2h). Per-protocol analyses revealed that patients who were adherent to the diet reported less complaints of fatigue, nausea and insomnia. There were no differences in the other symptom scales between adherent and non-adherent FMD patients or patients on a regular diet (supplementary material).

#### Functional scales of the QLQ-BR23

There were no differences between groups over time in the functional scales body image, sexual functioning and future perspective. Patients on a regular diet reported better scores on sexual enjoyment at the last time point, whereas patients on the FMD did not fully recover to baseline values (*p* = 0.040). In both arms, patients reported lower scores on body image, sexual functioning and sexual enjoyment during treatment (Fig. 3a–d). Per-protocol analyses did not show any differences between groups (supplementary material).

#### QLQ-BR23 symptom scales

The side effects of chemotherapy and hair loss scores worsened in both arms during treatment (Fig. 3e, f). There were no differences between groups in intention-to-treat or per-protocol analyses (supplementary material).

### Distress

**Fig. 4:**
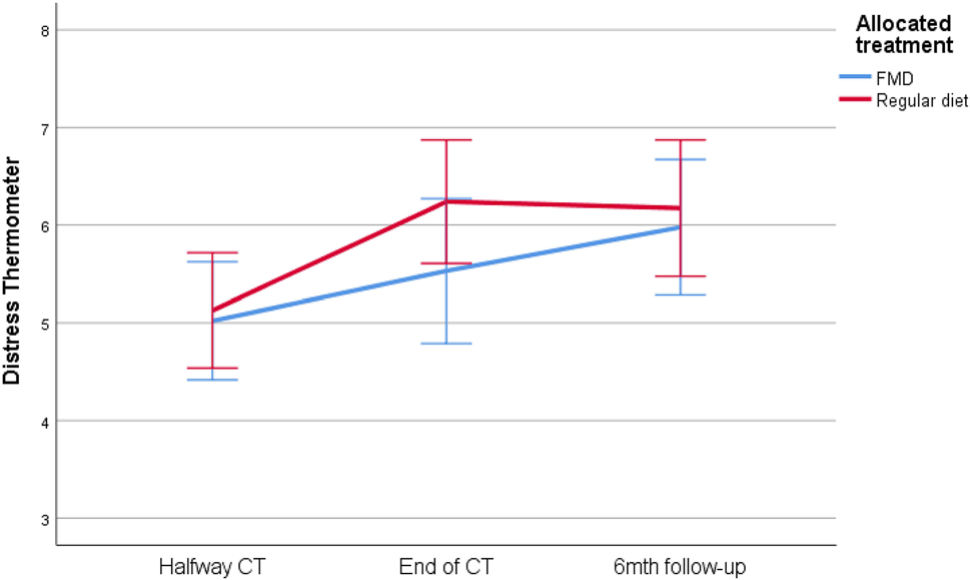
Distress thermometer. Psychosocial distress given for 3 time points: halfway therapy, at the end of therapy and at six months follow-up. Error bars indicate the 95% CI. *CT* chemotherapy, *FMD* fasting mimicking diet,* CI* confidence interval

The mean Distress Thermometer (DT) score halfway chemotherapy for all patients was 5.19 (SD = 2.1), with a range of 1–10. 61.3% of the patients experienced clinically relevant distress (DT score ≥5). During treatment and at 6-month follow-up, clinically relevant distress gradually increased in both groups to 78.0% and 70.7% of control and FMD patients, respectively (Fig. 4). There were no differences between groups in scores at the 3 time points, or over time (Table 4). The per-protocol analyses yielded similar results and did not uncover differences between groups over time (supplementary material).

### Illness perception

**Fig. 5:**
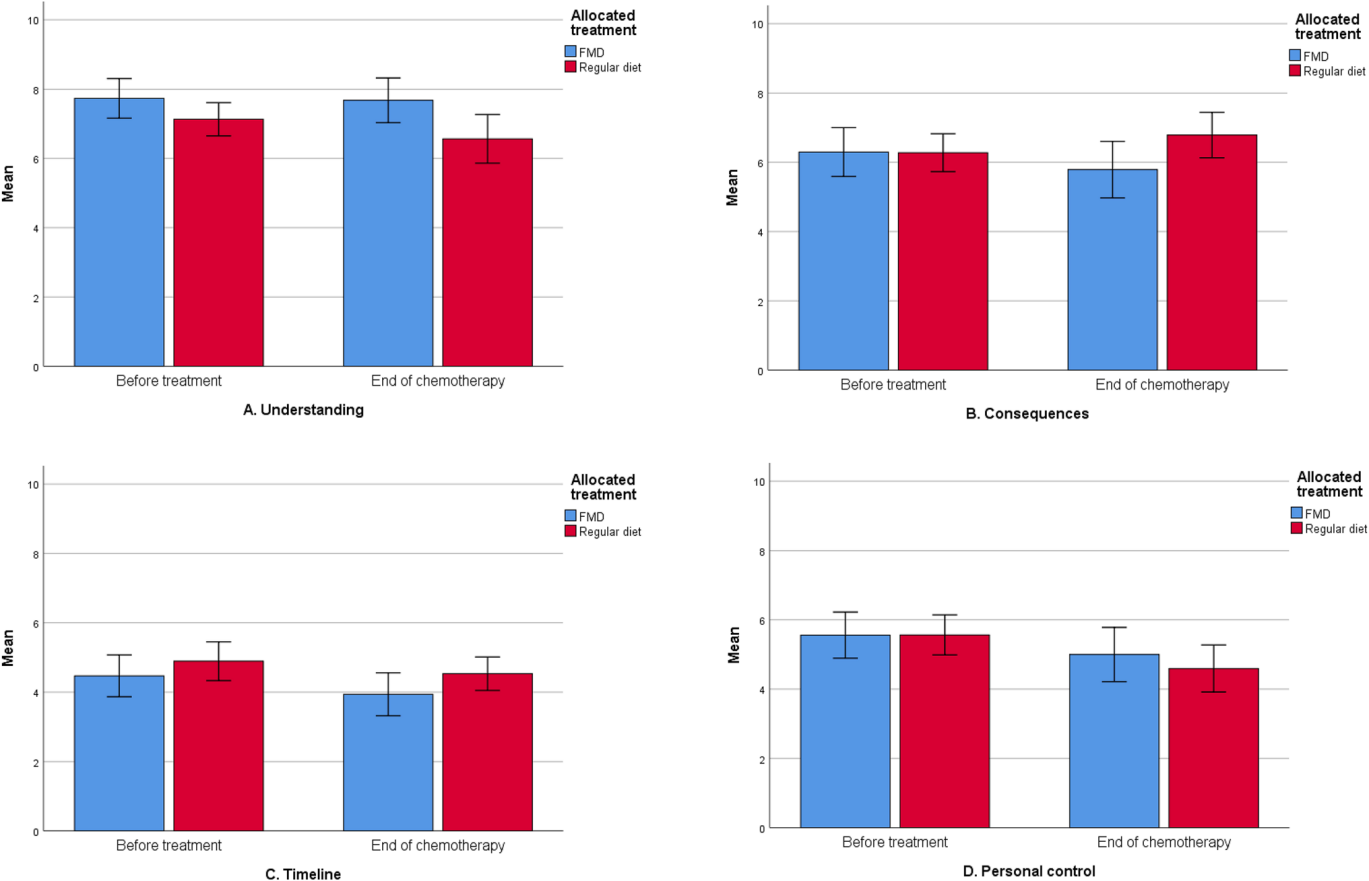

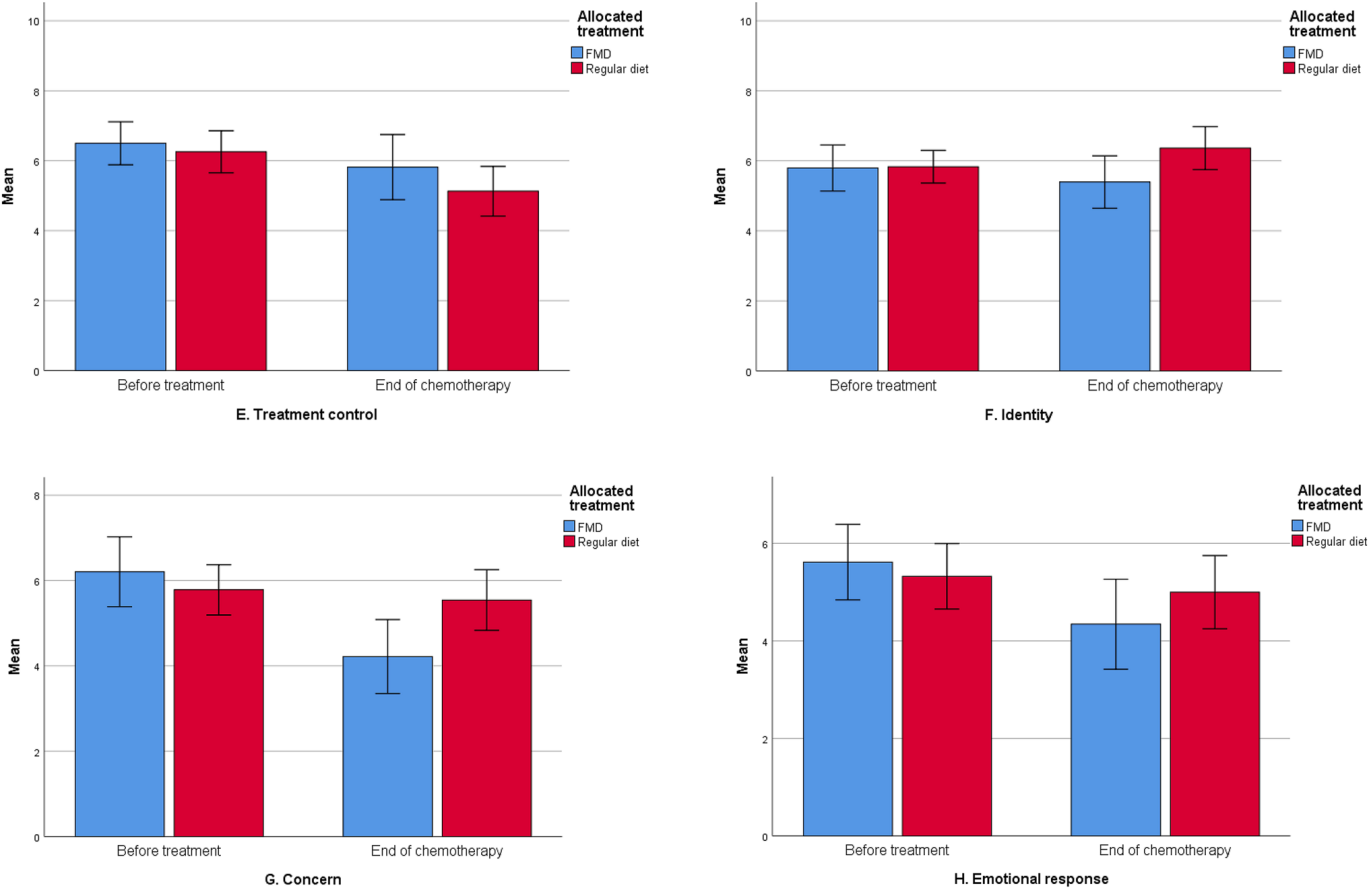
**a**–**h **Illness perceptions

At baseline, there were no different perceptions of the possible side effects of their treatment between groups (Table 3). Patients believed to have personal control over the possible side effects, were positive about the effectiveness of their treatment and felt they had a good understanding of potential adverse effects. At the end of chemotherapy FMD patients reported numerically but not statistically significant more positive outcomes of almost every perception, with the greatest improvement of concerns and emotional response (Fig. 5a–h; Table 5). In comparison with controls, FMD patients felt they had better understanding of side effects (*p* ≤ 0.01), and they were less concerned about them over time (*p* < 0.05, Table 5). In both groups, patients reported to believe they had less personal control over their side effects in the course of treatment (Fig. 5d). In the per-protocol analyses more positive perceptions of understanding, consequences (how much the side effects affect their life) and identity (how much side effects they experience) were observed in patients adherent to the FMD in comparison with patients on a regular diet (supplementary material).

**Table 5:** Mean scores of BIPQ by treatment arm

	Questionnaire time points		*p* value		
	Baseline	Before last cycle of chemotherapy	Time	Randomization	Time by randomization
BIPQ					
Understanding					
Regular diet	7.08 (1.84)	6.57 (2.36)	0.674	**0.009**	0.193
FMD	7.36 (1.85)	7.75 (1.78)			
Consequences					
Regular diet	6.41 (1.77)	6.79 (2.24)			
FMD	6.25 (1.97)	5.91 (2.38)	0.155	0.196	0.148
Timeline					
Regular diet	5.07 (1.83)	4.53 (1.64)	0.992	0.118	0.843
FMD	4.47 (1.77)	3.91 (1.73)			
Personal control					
Regular diet	5.62 (1.93)	4.60 (2.31)	**0.036**	0.536	0.540
FMD	5.37 (1.92)	5.00 (2.17)			
Treatment control					
Regular diet	6.34 (1.94)	5.13 (2.42)	0.060	0.203	0.475
FMD	6.43 (1.57)	5.82 (2.59)			
Identity					
Regular diet	6.03 (1.57)	6.36 (2.09)	0.081	0.072	0.234
FMD	5.90 (1.78)	5.53 (2.22)			
Concern					
Regular diet	6.05 (2.03)	5.54 (2.39)	0.758	0.230	**0.033**
FMD	6.08 (2.56)	4.24 (2.37)			
Emotional response					
Regular diet	6.05 (2.03)	5.00 (2.53)	0.922	0.489	0.145
FMD	5.90 (2.09)	4.30 (2.53)			

*P*-values < 0.05 were considered significant

*P time* changes of Illness perception scores over time, *P randomization* differences in Illness perceptions between the two treatment groups, *P time by randomization* different effects between treatment groups over time, *FMD *fasting mimicking diet

## Discussion

The randomized, phase 2 DIRECT trial demonstrated no impact of an FMD as compared with a regular diet on grade III/IV toxicity, as documented by a physician and graded according to the Common Terminology Criteria for Adverse Events version 4.03, during neoadjuvant chemotherapy in patients with HER2-negative breast cancer [8]. The current analysis indicates that, patient-reported outcome measures (PROMs) were distinct in some respects between groups. Our data suggest that the FMD was associated with increased overall well-being from a patients’ perspective. A per-protocol analysis, yielding better scores of various aspects of QoL in patients who were adherent to the diet than in those who were not, or in controls, supports this inference.

As expected, neoadjuvant chemotherapy was accompanied by the occurrence of side effects, impaired QoL and distress, with recovery of most of the scores after 6 months of follow-up. This is in line with previous studies of patients with breast cancer receiving anthracycline- and taxane-based regimens of chemotherapy [9, 10, 31, 32]. The mean baseline EORTC-QLQ-C30 and QLQ-BR23 scores of patients in our study were largely similar to the reference values for patients with early stage breast cancer [33]. Also, the illness perceptions before the start of chemotherapy were quite similar to those reported in other patients with breast cancer [34], although the perception of personal control in our study seemed slightly stronger than usually reported. Perhaps patients who gave informed consent for the trial became convinced that an FMD could ameliorate the side effects of chemotherapy after reading the study information.

FMD patients did not score worse than controls on any of the subscales of the EORTC-QLQ-C30, the QLQ-BR23, or the distress thermometer. In fact, post hoc analyses revealed that patients who were adherent to the diet (at least half of the cycles of chemotherapy) had favorable outcomes regarding physical, role, emotional, cognitive and social functioning, and had fewer complaints of fatigue, nausea and insomnia than non-adherent patients or controls. These positive effects of the FMD are in line with other trials and animal studies, in which STF and FMD enhanced cognitive performance [7], improved QoL [22, 35] and reduced fatigue [22] in patients with cancer and people with the metabolic syndrome. In addition to metabolic benefits, the periodic fasting mimicking diet seems to have beneficial effects on patients’ overall well-being and functioning in daily life.

Although the effects of fasting on treatment with chemotherapy in patients with cancer are currently uncertain, promising results of preclinical and clinical studies, extensively covered by the media, spurred enthusiasm for fasting among patients with cancer [24]. Fasting made people feel more proactively involved in treatment and recovery [24]. We did not observe such differences in our illness perception measures, as both treatment groups were positive about their personal control and the effectiveness of their treatment. We did find that FMD patients had better understanding and were less concerned about the possible side effects in the course of their treatment. A meta-analysis of the BIPQ showed associations between negative concern perceptions and lower scores on QoL assessments on psychological, physical and fatigue domains [12]. The results of our per-protocol analysis with more positive outcomes on similar QoL domains in patients adherent to the FMD are in line with this finding.

In general, patients with breast cancer gain weight during chemotherapy, which often persists in the years following completion of treatment [36–38]. Weight gain can lead to poor QoL, physiological stress and body image issues [39, 40]. Reduction in physical activity, dietary changes, the use of steroids as anti-emetics, and therapy-induced menopause all contribute to weight gain during breast cancer treatment. In our trial, patients in the FMD group were not prescribed dexamethasone prior to AC/FEC, because we previously found that this prevents the decline of glucose and insulin in response to the diet [41]. Although patients were not allowed to lose more than 10% of bodyweight during the trial, patients using the FMD had a moderate decrease in body mass index (BMI) during the course of treatment, which was not seen in patients on a regular diet. At 6 months of follow-up patients on the FMD had maintained their normal weight, while patients who followed their regular diet displayed an increase in BMI. Thus, an FMD may offer protection against the common weight gain during and after treatment with chemotherapy in patients with breast cancer.

This study has some limitations. Although the percentage of returned questionnaires was high, questionnaire completion declined in the course of the trial period, and completion rates differed between groups. In particular, patients who were non-adherent to the FMD more often failed to fill out the last two questionnaires. This might provide biased results, as it is conceivable that patients who stopped the FMD because of side effects would have reported lower scores on QoL as well. Furthermore, the lack of blinding, which is obviously very difficult in any nutrition trial, may have affected patients ‘behavior and perceptions. Finally, it is important to point out that the results of our per-protocol analyses should be cautiously interpreted. In particular, it is conceivable that patients who felt better for any reason were more inclined to stick to their dietary prescriptions, which would dismiss the putative benefits of the FMD for well-being, in defiance of the myriad indications to the contrary in previous studies [7, 22, 35].

To our knowledge, this is the first large randomized trial assessing the effect of an FMD on QoL in patients with breast cancer. Our results need to be confirmed in other trials, which are currently ongoing. Furthermore, we plan to do more research to improve adherence to short-term fasting and FMDs, using revised diets.

In conclusion, our study suggests that an FMD as an adjunct to neoadjuvant chemotherapy may have beneficial effects on certain QoL and illness perceptions domains in patients with HER2-negative breast cancer, which is in line with previous reports on clinical response and safety.

## Electronic supplementary material

Below is the link to the electronic supplementary material.

Supplementary file1 (DOCX 315 kb)
