# Supplementary material for: Quality of life and illness perceptions in patients with breast cancer using a fasting mimicking diet as an adjunct to neoadjuvant chemotherapy in the phase 2 DIRECT (BOOG 2013–14) trial
Source: Breast Cancer Res Treat. 2020 Nov 11;185(3):741–58. doi: 10.1007/s10549-020-05991-x (PMC7921018; doi:10.1007/s10549-020-05991-x)
Supplement: Supplementary file 1 — Supplementary file1 (DOCX 315 kb) [file 10549_2020_5991_MOESM1_ESM.docx]

**Supplementary material**

*Table S1: Mean scores of QLQ-C30 and QLQ-BR23 functioning scales and symptom ratings and Distress thermometer for patients on a regular diet, patients adherent to the FMD and patients that were not adherent to the FMD (per protocol analysis)*

|  |  | **Questionnaire time points** | | | | **p-value** | | |
| --- | --- | --- | --- | --- | --- | --- | --- | --- |
|  |  | **Baseline** | **Halfway CT** | **Before last cycle of CT** | **6-months after surgery** | **Time** | **Randomisation** | **Time by Randomisation** |
| **EORTC QLQ-C30**  Regular diet (n=53)  Adherent (n=21)  Non-adherent (n=33) | | | | | | | | |
| Global Health | Regular diet  Adherent  Non-adherent | 80.5  80.6  78.8 | 70.3  75.4  68.6 | 59.2  62.8  60.5 | 75.8  79.8  77.8 | **<0.001** | 0.104 | 0.959 |
| **Functioning scales** |  |  |  |  |  |  |  |  |
| Physical functioning | Regular diet  Adherent  Non-adherent | 93.7  96.5  96.4 | 81.9  88.6  81.2 | 68.8  80.0  74.6 | 82.8  88.1  84.5 | **<0.001** | **0.040** | 0.649 |
| Role functioning | Regular diet  Adherent  Non-adherent | 90.1  88.9  85.3 | 66.1  82.5  57.4 | 52.4  58.9  48.2 | 74.1  81.7  75.0 | **<0.001** | **0.022** | 0.166 |
| Emotional functioning | Regular diet  Adherent  Non-adherent | 75.3  73.0  68.0 | 80.7  86.1  76.5 | 73.4  83.3  74.6 | 78.1  87.7  73.3 | **0.041** | **0.010** | 0.883 |
| Cognitive Functioning | Regular diet  Adherent  Non-adherent | 89.4  86.5  82.8 | 77.2  85.7  77.0 | 70.5  74.4  76.3 | 76.9  83.3  67.4 | **0.009** | **0.013** | 0.449 |
| Social functioning | Regular diet  Adherent  Non-adherent | 92.0  92.1  86.4 | 74.9  88.9  72.5 | 62.8  72.2  65.8 | 83.6  90.5  77.1 | **0.008** | **0.039** | 0.813 |
| **Symptoms** |  |  |  |  |  |  |  |  |
| Fatigue | Regular diet  Adherent  Non-adherent | 19.0  14.8  28.1 | 39.8  22.2  49.8 | 52.8  39.3  46.8 | 31.1  25.4  31.9 | **<0.001** | **0.007** | **0.019** |
| Nausea | Regular diet  Adherent  Non-adherent | 4.3  5.6  3.4 | 15.5  4.8  16.7 | 5.9  0.0  13.2 | 2.5  1.2  4.7 | **<0.001** | **0.004** | 0.163 |
| Pain | Regular diet  Adherent  Non-adherent | 11.5  6.3  8.3 | 15.8  4.0  11.3 | 22.9  21.1  28.9 | 17.3  11.1  27.8 | **<0.001** | 0.303 | 0.270 |
| Dyspnea | Regular diet  Adherent  Non-adherent | 4.6  6.3  2.0 | 14.0  12.7  25.5 | 26.4  28.9  14.0 | 16.0  6.3  12.5 | **0.001** | 0.302 | **0.004** |
| Insomnia | Regular diet  Adherent  Non-adherent | 26.4  31.7  33.3 | 26.9  22.2  25.5 | 37.5  31.1  45.6 | 38.3  20.6  40.3 | **<0.001** | **0.048** | 0.067 |
| Appetite loss | Regular diet  Adherent  Non-adherent | 9.8  11.1  16.7 | 17.5  9.5  21.6 | 29.9  22.2  21.1 | 7.4  3.2  6.9 | **<0.001** | 0.083 | 0.613 |
| Constipation | Regular diet  Adherent  Non-adherent | 6.9  3.2  5.9 | 25.1  17.5  26.5 | 22.2  13.3  19.3 | 8.0  9.5  6.9 | **<0.001** | 0.461 | 0.700 |
| Diarrhea | Regular diet  Adherent  Non-adherent | 2.3  4.8  1.0 | 7.0  4,8  8.8 | 10.4  4.4  21.1 | 1.2  1.6  2.8 | **0.001** | 0.283 | 0.246 |
| Financial difficulties | Regular diet  Adherent  Non-adherent | 2.9  1.6  5.2 | 9.9  3.2  11.8 | 11.8  6.7  5.3 | 6.2  9.5  18.1 | 0.790 | 0.555 | 0.051 |
| **EORTC QLQ-BR23**  Regular diet (n=53)  Adherent (n=21)  Non-adherent (n=33) | | | | | | | | |
| Body image | Regular diet  Adherent  Non-adherent | 89.9  91.3  89.7 | 78.8  72.5  73.6 | 65.1  69.4  67.5 | 78.4  75.8  72.9 | **<0.001** | 0.756 | 0.632 |
| Sexual functioning | Regular diet  Adherent  Non-adherent | 77.0  81.0  73.0 | 81.9  79.4  77.9 | 86.8  86.7  86.0 | 73.0  75.8  75.7 | **<0.001** | 0.604 | 0.604 |
| Sexual enjoyment | Regular diet  Adherent  Non-adherent | 58.1  46.2  61.3 | 44.4  47.2  50.7 | 43.1  33.3  50.0 | 55.9  45.5  51.1 | **0.001** | 0.849 | 0.133 |
| Future perspective | Regular diet  Adherent  Non-adherent | 42.0  49.2  47.1 | 34.5  33.3  44.1 | 45.8  37.8  36.8 | 40.7  33.3  43.1 | 0.163 | 0.087 | 0.248 |
| **Symptom scales** |  |  |  |  |  |  |  |  |
| Side-effects  of systemic therapy | Regular diet  Adherent  Non-adherent | 10.5  8.9  10.1 | 32.8  26.8  35.0 | 40.0  36.1  35.7 | 18.5  15.5  17.3 | **<0.001** | 0.164 | 0.523 |
| Upset by  hair loss | Regular diet  Adherent  Non-adherent | 16.7  33.3  33.3 | 32.6  38.6  35.6 | 31.1  41.7  22.2 | 50.0  44.4  33.3 | **0.003** | 0.529 | 0.575 |
| **Distress thermometer**  Regular diet (n=53)  Adherent (n=21)  Non-adherent (n=18) | | | | | | | | |
|  | Regular diet  Adherent  Non-adherent |  | 5.21  3.95  5.91 | 6.27  5.07  5.89 | 6.17  4.86  6.96 | **0.450** | 0.086 | 0.207 |

***P time: changes of QoL scores over time***

***P randomisation: differences in QoL between patients adherent to FMD and patients on a regular diet***

***P time by randomisation*: different effects between treatment groups over time**

*Abbreviations: CT chemotherapy, FMD Fasting mimicking diet*


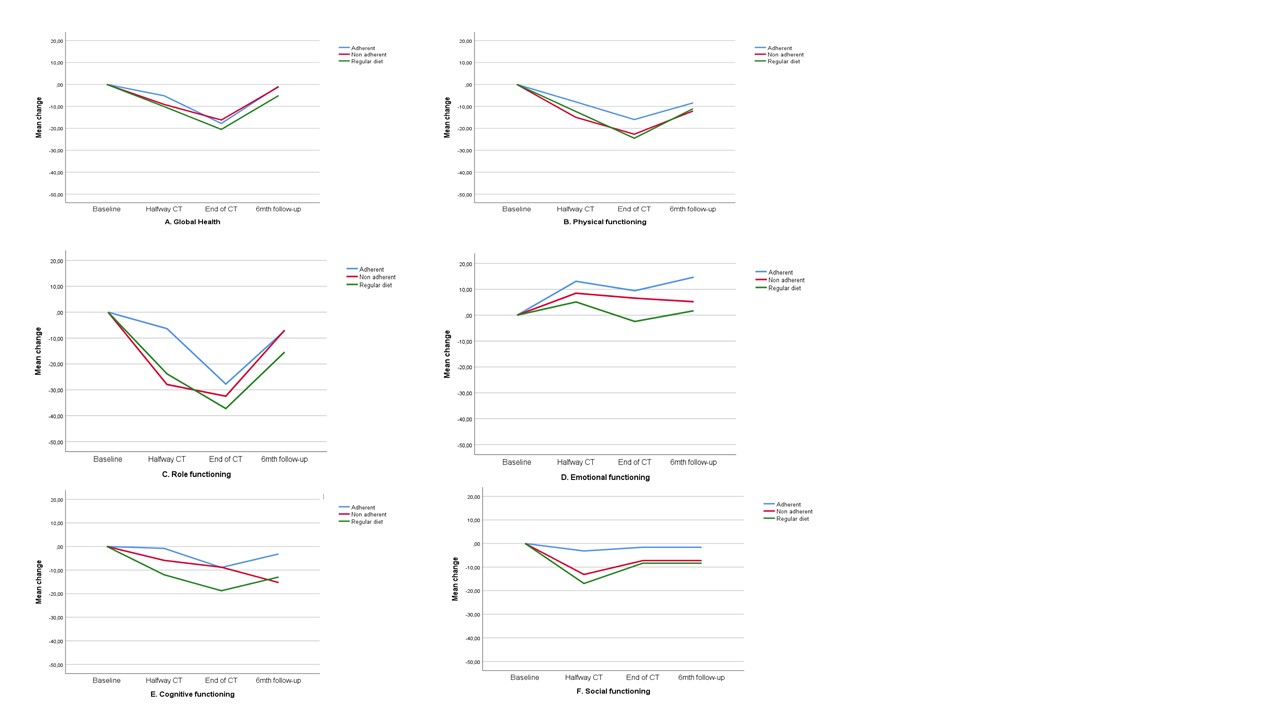
*Figure S1A-O: Mean changes from baseline on functional and symptom scales of the EORTC-QLQ C30 for patients on a regular diet, patients adherent to the FMD and patients that were not adherent to the FMD (per protocol analysis)*


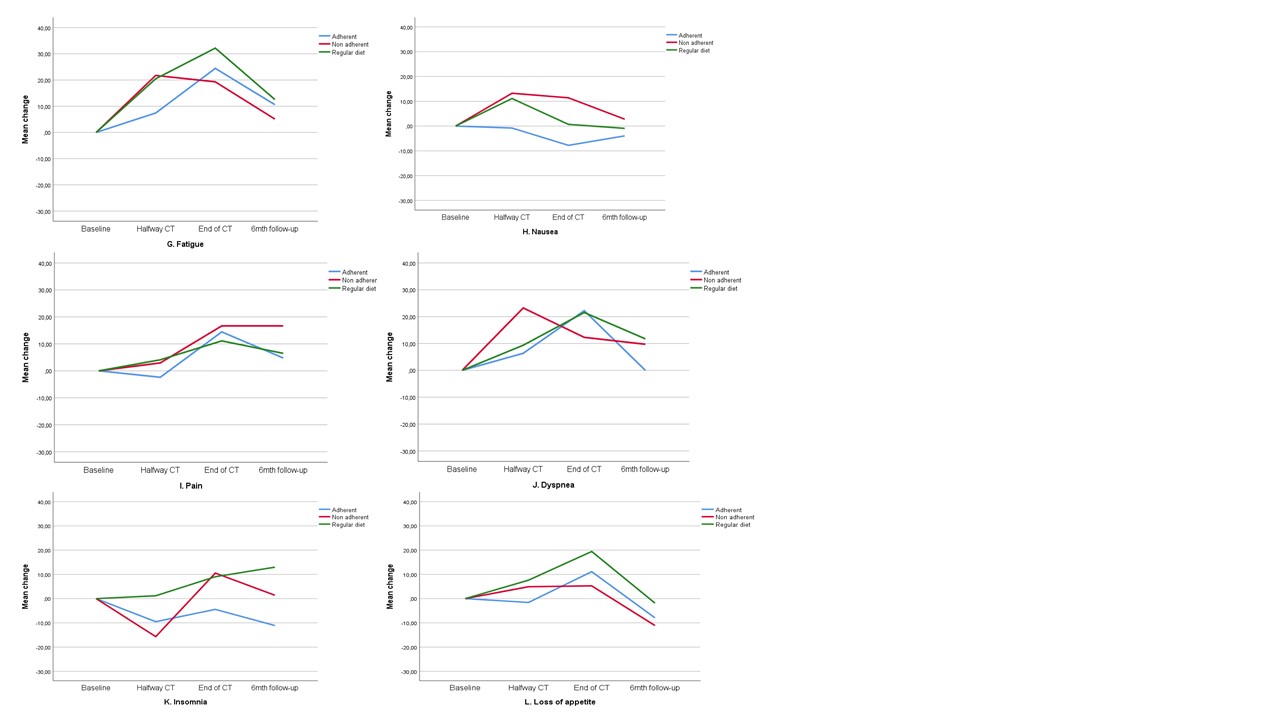


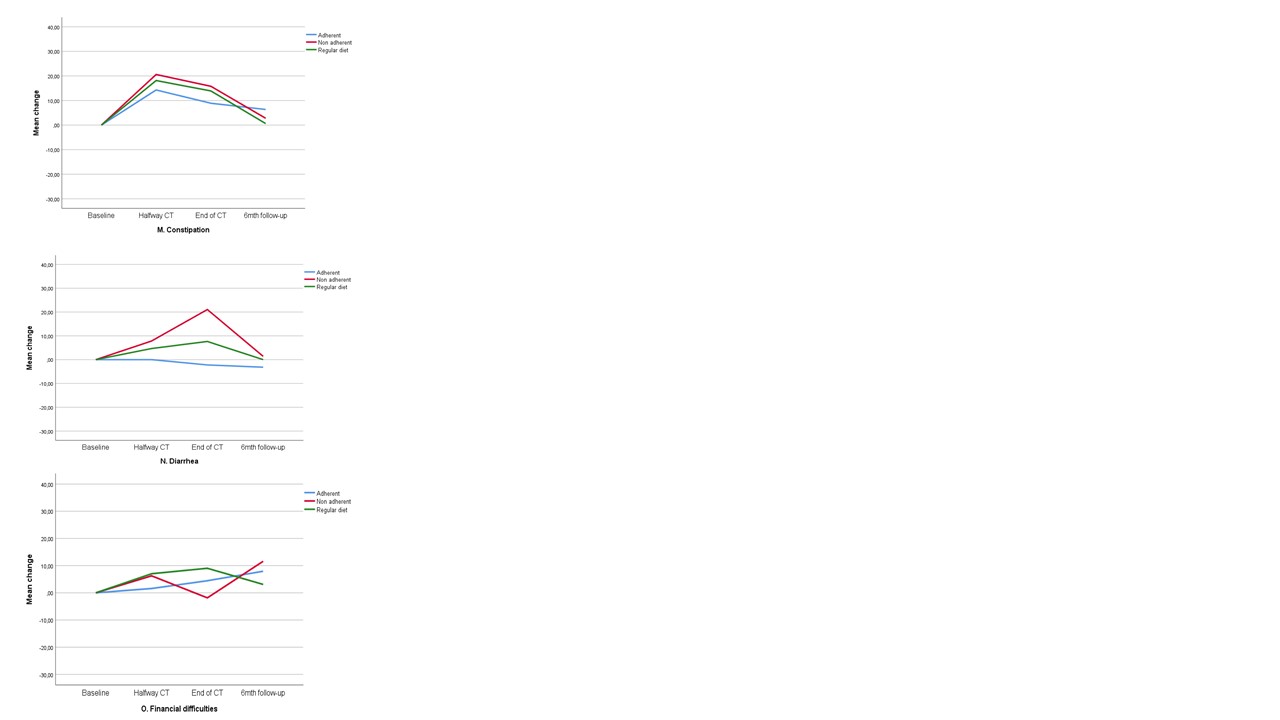


*These plots show mean changes calculated from the raw data; they are not model estimates, and they are not adjusted for any covariates.*  *Lower scores on the functional scales (fig. A-F) implicates lower quality of life, lower scores on the symptom scales (fig. G-O) implicate better quality of life*

*Abbreviations: CT chemotherapy; FMD Fasting mimicking diet; CI: confidence interval*


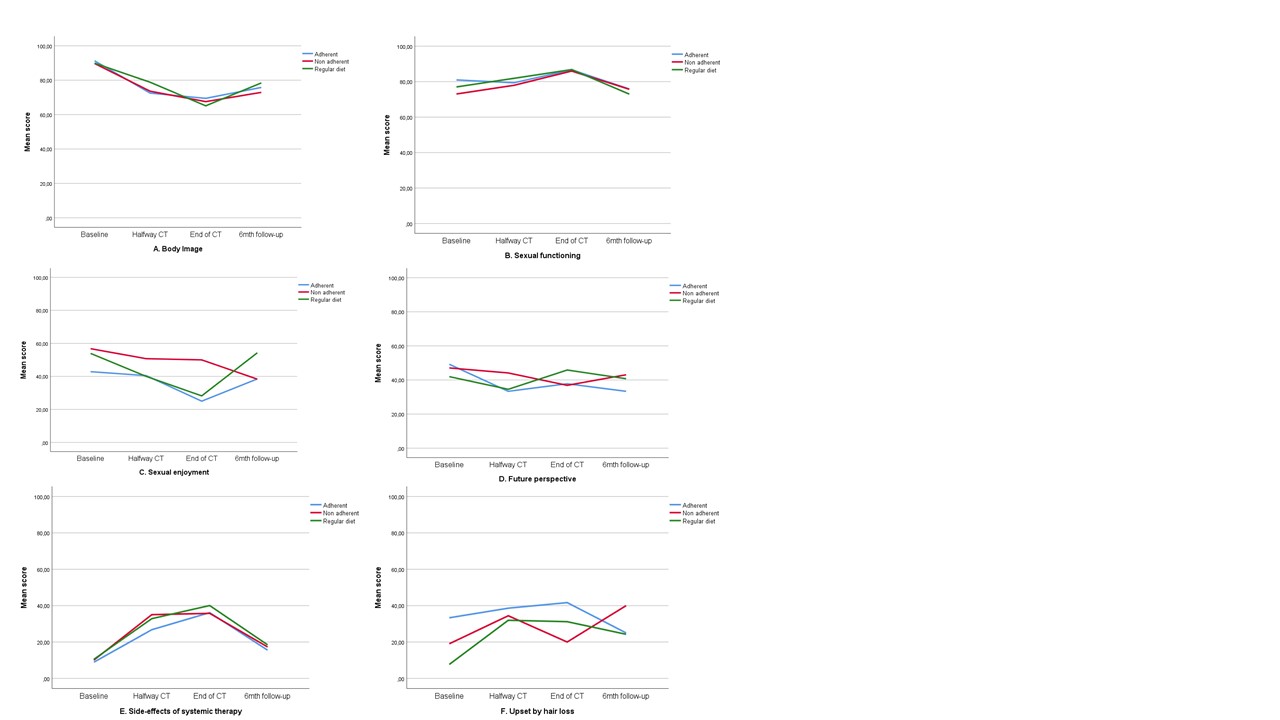
*Figure S2A-F: Mean scores on functional and symptom scales of the EORTC QLQ-BR23 for patients on a regular diet, patients adherent to the FMD and patients that were not adherent to the FMD (per protocol analysis)*

*These plots show mean scores calculated from the raw data; they are not model estimates, and they are not adjusted for any covariates. Lower scores on the functional scales (fig. A-D) implicates lower quality of life, lower scores on the symptom scales (fig. E and F) implicate better quality of life*

*Abbreviations: CT chemotherapy; FMD Fasting mimicking diet; CI: confidence interval*

*Figure S3 Distress thermometer (per protocol analysis)*


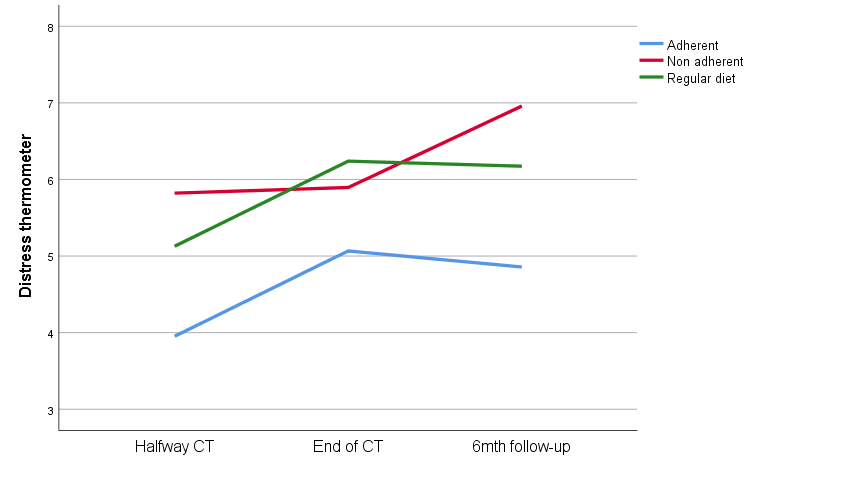


*Psychosocial distress given for 3 timepoints: halfway therapy, at the end of therapy and at six months follow-up.*

*Abbreviations: CT chemotherapy; FMD: fasting mimicking diet;*

*Table S2: BIPQ Scores (means and standard deviations) between patients on a regular diet and patients adherent to the FMD (per protocol analysis)*

|  |  | **Questionnaire time points** | | **p-value** | | |
| --- | --- | --- | --- | --- | --- | --- |
|  |  | **Baseline** | **Before last cycle of chemotherapy** | **Time** | **Randomisation** | **Time by Randomisation** |
| **BIPQ**  Understanding | Regular diet (n=40)  Adherent (n=14) | 7,08 (1,84)  7,14 (2,08) | 6.57 (2.36)  7.86 (1.29) | 0.841 | **0.044** | 0.320 |
| Consequences | Regular diet (n=42)  Adherent (n=15) | 6,41 (1,77)  6,00 (2,07) | 6.79 (2.24)  5.27 (1.83) | 0.827 | **0.048** | 0.111 |
| Timeline | Regular diet (n=42)  Adherent (n=15) | 5,07 (1,83)  4,29 (1,45) | 4.53 (1.64)  4.53 (1.64) | 0.911 | 0.466 | 0.491 |
| Personal control | Regular diet (n=41)  Adherent(n=15) | 4,38 (1,93)  4,71 (1,82) | 5.40 (2.31)  4.93 (2.34) | 0.159 | 0.453 | 0.793 |
| Treatment control | Regular diet (n=42)  Adherent (n=15) | 3,66 (1,94)  3,30 (1,78) | 4.87 (2.42)  3.53 (2.53) | 0.262 | 0.117 | 0.315 |
| Identity | Regular diet (n=42)  Adherent(n=15) | 6,03 (1,57)  5,33 (1,56) | 6.36 (2.09)  4.80 (1.74) | 0.695 | **0.013** | 0.132 |
| Concern | Regular diet (n=40)  Adherent (n=14) | 6,05 (2,03)  5,67 (1,91) | 5.54 (2.39)  4.50 (2.03) | 0.774 | 0.278 | 0.242 |
| Emotional response | Regular diet (n=40)  Adherent (n=14) | 5,58 (2,09)  5,43 (1,86) | 5.00 (2.53)  3.79 (2.49) | 0.292 | 0.198 | 0.201 |

***P time: changes of Illness perception scores over time***

***P randomisation: differences in Illness perceptions between patients adherent to FMD and patients on a regular diet***

***P time by randomisation*: different effects between treatment groups over time**

*Figure S4 Chemolieve^TM^ fasting mimicking diet schedule.*
